# 6-Bromo-2-naphthol–piperazine (2/1)

**DOI:** 10.1107/S1600536808036878

**Published:** 2008-11-13

**Authors:** Yan Tian, Deliang Cui

**Affiliations:** a State Key Laboratory of Crystalline Materials, Shandong University, Jinan 250100, People’s Republic of China and School of Chemistry & Chemical Engineering, Shandong University, Jinan 250100, People’s Republic of China

## Abstract

In the title compound, 2C_10_H_7_BrO·C_4_H_10_N_2_, the piperazine (pip) mol­ecule displays a chair conformation and is linked to two mol­ecules of 6-bromo-2-naphthol (bno) *via* O—H⋯N hydrogen bonds. Weak N—H⋯O hydrogen bonds from pip to bno mol­ecules result in chains propagating in [100]. The chains inter­act via C—H⋯π inter­actions.

## Related literature

For related structures, see: Wang & Tang (2006*a* ▶,*b* ▶,*c* ▶); Wang *et al.* (2008 ▶).
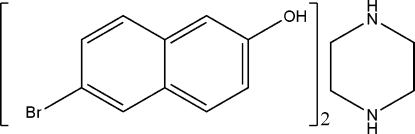


## Experimental

### 

#### Crystal data

2C_10_H_7_BrO·C_4_H_10_N_2_
*M* *_r_* = 532.27
Monoclinic, 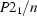

*a* = 10.1327 (4) Å
*b* = 16.2494 (7) Å
*c* = 14.3499 (5) Å
β = 108.238 (2)°
*V* = 2244.02 (15) Å^3^
*Z* = 4
Mo *K*α radiation
μ = 3.63 mm^−1^
*T* = 296 (2) K
0.30 × 0.30 × 0.10 mm

#### Data collection

Bruker SMART CCD diffractometer
Absorption correction: multi-scan (*SADABS*; Bruker, 2001 ▶) *T*_min_ = 0.409, *T*_max_ = 0.713
16751 measured reflections
5164 independent reflections
2857 reflections with *I* > 2σ(*I*)
*R*_int_ = 0.035

#### Refinement

*R*[*F* ^2^ > 2σ(*F* ^2^)] = 0.041
*wR*(*F* ^2^) = 0.107
*S* = 1.00
5164 reflections
271 parameters
H-atom parameters constrained
Δρ_max_ = 0.52 e Å^−3^
Δρ_min_ = −0.39 e Å^−3^

### 

Data collection: *SMART* (Bruker, 2001 ▶); cell refinement: *SAINT* (Bruker, 2001 ▶); data reduction: *SAINT*; program(s) used to solve structure: *SHELXS97* (Sheldrick, 2008 ▶); program(s) used to refine structure: *SHELXL97* (Sheldrick, 2008 ▶); molecular graphics: *SHELXTL* (Sheldrick, 2008 ▶); software used to prepare material for publication: *SHELXTL*.

## Supplementary Material

Crystal structure: contains datablocks global, I. DOI: 10.1107/S1600536808036878/hb2836sup1.cif

Structure factors: contains datablocks I. DOI: 10.1107/S1600536808036878/hb2836Isup2.hkl

Additional supplementary materials:  crystallographic information; 3D view; checkCIF report

## Figures and Tables

**Table 1:** Hydrogen-bond geometry (Å, °)

*D*—H⋯*A*	*D*—H	H⋯*A*	*D*⋯*A*	*D*—H⋯*A*
O1—H1*B*⋯N1	0.82	1.94	2.743 (4)	168
O2—H2*B*⋯N2^i^	0.83	1.88	2.694 (4)	163
N1—H1*C*⋯O2	0.83	2.47	3.235 (4)	152
N2—H2*A*⋯O1^ii^	0.77	2.50	3.184 (4)	149
C4—H4*A*⋯*Cg*5	0.93	2.77	3.471 (3)	133
C14—H14*A*⋯*Cg*2^iii^	0.93	2.68	3.371 (3)	132
C16—H16*A*⋯*Cg*1^iii^	0.93	2.90	3.570 (3)	130
C21—H21*A*⋯*Cg*2^iv^	0.97	2.93	3.831 (3)	156

Symmetry codes: (i) 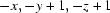; (ii) 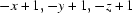; (iii) 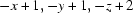; (iv) 
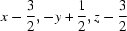
. *Cg*1, *Cg*2 and *Cg*5 are the centroids of atoms C1–C5,C10, C5–C10 and C15–C20, respectively.

**Fig. 1.**
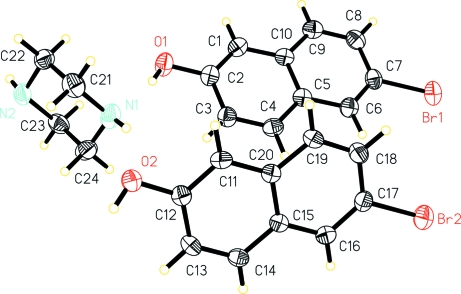
A drawing of (I), with displacement ellipsoids for the non-hydrogen atoms drawn at the 30% probability level.

**Fig. 2.**
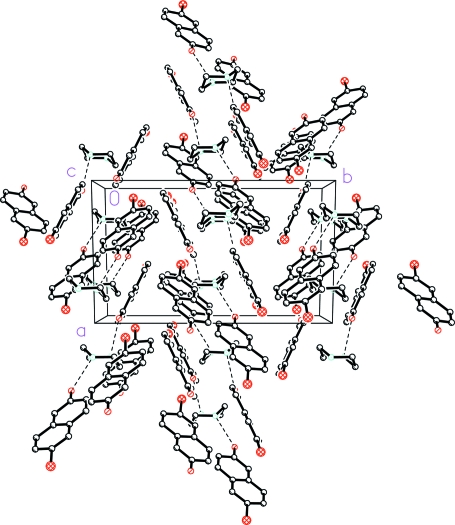
Packing diagram of (I); hydrogen bonds are shown by dashed lines.

## supplementary crystallographic information

### Comment

During the past decade, the field of molecular co-crystals have received considerable attention, for example, the design, construction and properties of molecular co-crystals. Recently, many co-crystals containing some organic acids and bases, have been successfully prepared and characterized by some research groups (Wang *et al.*, 2006a,b,c). Especially, co-crystals containing hydroxyl-naphthalene with some organic bases have been synthesized and characterized (Wang *et al.*, 2008). As part of our investigations of co-crystals containing 6-bromo-2-naphthol (bno), we now report the structure of the co-crystal, (I), of bno and piperazine.

A view of the title structure is shown in Fig. 1. The asymmetric unit consists of two independent bno molecules and one independent molecule of piperazine. In the crystal structure of (I), the piperazine molecule display a chair conformation and links with two molecules of 6-bromo-2-naphthol *via* O—H···N hydrogen bonds. These motifs are extended to one-dimensional chains *via* intermolecular edge-to-face C—H···π packing interactions (Fig. 2 and Table 1).

### Experimental

A mixture of bno (446 mg, 1 mmol) and piperazine (86 mg, 1 mmol) was dissolved in methanol (10 ml), which was left at room temperature. Some colourless plates of (I0 were obtained after ten days. Analysis found (%): C, 54.28; H, 4.53; N, 5.28; requires (%): C, 54.16; H, 4.54; N, 5.26.

### Refinement

All the H atoms were located in a difference Fourier map. The carbon-bound hydrogen atoms were relocated to idealised positions (C—H = 0.93 A °), and refined as riding with *U*_iso_(H) = 1.2U_eq_(C). The oxygen- and nitrogen-bound hydrogen atoms were refined as riding in their as-found relative possitions with *U*_iso_(H) = 1.5U_eq_(O, N).

### Crystal data

**Table:**

2C_10_H_7_BrO·C_4_H_10_N_2_	*F*_000_ = 1072
*M**_r_* = 532.27	*D*_x_ = 1.575 Mg m^−^^3^
Monoclinic, *P*2_1_/*n*	Mo *K*α radiation λ = 0.71073 Å
Hall symbol: -P 2yn	Cell parameters from 3655 reflections
*a* = 10.1327 (4) Å	θ = 2.5–27.5º
*b* = 16.2494 (7) Å	µ = 3.64 mm^−^^1^
*c* = 14.3499 (5) Å	*T* = 296 (2) K
β = 108.238 (2)º	Plate, colourless
*V* = 2244.02 (15) Å^3^	0.30 × 0.30 × 0.10 mm
*Z* = 4	

### Data collection

**Table:**

Bruker SMART CCD diffractometer	5164 independent reflections
Radiation source: fine-focus sealed tube	2857 reflections with *I* > 2σ(*I*)
Monochromator: graphite	*R*_int_ = 0.035
Detector resolution: 9.00 pixels mm^-1^	θ_max_ = 27.6º
*T* = 296(2) K	θ_min_ = 2.0º
ω scans	*h* = −13→13
Absorption correction: multi-scan(SADABS; Bruker, 2001)	*k* = −20→21
*T*_min_ = 0.409, *T*_max_ = 0.713	*l* = −18→18
16751 measured reflections	

### Refinement

**Table:**

Refinement on *F*^2^	Secondary atom site location: difference Fourier map
Least-squares matrix: full	Hydrogen site location: inferred from neighbouring sites
*R*[*F*^2^ > 2σ(*F*^2^)] = 0.041	H-atom parameters constrained
*wR*(*F*^2^) = 0.107	*w* = 1/[σ^2^(*F*_o_^2^) + (0.046*P*)^2^ + 0.5753*P*] where *P* = (*F*_o_^2^ + 2*F*_c_^2^)/3
*S* = 1.00	(Δ/σ)_max_ = 0.001
5164 reflections	Δρ_max_ = 0.52 e Å^−^^3^
271 parameters	Δρ_min_ = −0.39 e Å^−^^3^
Primary atom site location: structure-invariant direct methods	Extinction correction: none

### Special details

**Table:**

Geometry. All e.s.d.'s (except the e.s.d. in the dihedral angle between two l.s. planes) are estimated using the full covariance matrix. The cell e.s.d.'s are taken into account individually in the estimation of e.s.d.'s in distances, angles and torsion angles; correlations between e.s.d.'s in cell parameters are only used when they are defined by crystal symmetry. An approximate (isotropic) treatment of cell e.s.d.'s is used for estimating e.s.d.'s involving l.s. planes.
Refinement. Refinement of *F*^2^ against ALL reflections. The weighted *R*-factor *wR* and goodness of fit *S* are based on *F*^2^, conventional *R*-factors *R* are based on *F*, with *F* set to zero for negative *F*^2^. The threshold expression of *F*^2^ > σ(*F*^2^) is used only for calculating *R*-factors(gt) *etc*. and is not relevant to the choice of reflections for refinement. *R*-factors based on *F*^2^ are statistically about twice as large as those based on *F*, and *R*- factors based on ALL data will be even larger.

### Fractional atomic coordinates and isotropic or equivalent isotropic displacement parameters (Å^2^)

**Table:**

	*x*	*y*	*z*	*U*_iso_*/*U*_eq_	
Br1	0.90393 (4)	0.69713 (3)	1.25794 (3)	0.07368 (16)	
O1	0.5253 (2)	0.59439 (15)	0.67377 (16)	0.0644 (7)	
H1B	0.4423	0.5842	0.6586	0.097*	
C1	0.6945 (3)	0.6429 (2)	0.8149 (2)	0.0513 (8)	
H1A	0.7491	0.6579	0.7762	0.062*	
C2	0.5696 (3)	0.6067 (2)	0.7733 (2)	0.0500 (8)	
C3	0.4883 (3)	0.5817 (2)	0.8315 (2)	0.0528 (8)	
H3A	0.4037	0.5556	0.8023	0.063*	
C4	0.5317 (3)	0.59511 (19)	0.9301 (2)	0.0521 (8)	
H4A	0.4763	0.5783	0.9674	0.062*	
C5	0.6582 (3)	0.63370 (18)	0.9756 (2)	0.0452 (8)	
C6	0.7088 (3)	0.64743 (19)	1.0793 (2)	0.0520 (8)	
H6A	0.6553	0.6318	1.1184	0.062*	
C7	0.8328 (3)	0.6827 (2)	1.1201 (2)	0.0518 (8)	
C8	0.9161 (3)	0.70853 (19)	1.0628 (3)	0.0567 (9)	
H8A	1.0010	0.7341	1.0924	0.068*	
C9	0.8716 (3)	0.6958 (2)	0.9645 (3)	0.0558 (9)	
H9A	0.9273	0.7122	0.9272	0.067*	
C10	0.7427 (3)	0.65831 (18)	0.9179 (2)	0.0445 (7)	
C21	0.1839 (3)	0.6015 (2)	0.5035 (3)	0.0640 (10)	
H21A	0.1860	0.6602	0.5158	0.077*	
H21B	0.0877	0.5847	0.4757	0.077*	
C22	0.2618 (3)	0.5824 (2)	0.4333 (2)	0.0560 (9)	
H22A	0.2186	0.6104	0.3715	0.067*	
H22B	0.3567	0.6020	0.4597	0.067*	
C23	0.3232 (3)	0.4493 (2)	0.5094 (2)	0.0601 (9)	
H23A	0.4194	0.4658	0.5383	0.072*	
H23B	0.3209	0.3905	0.4974	0.072*	
C24	0.2437 (4)	0.4687 (3)	0.5782 (3)	0.0710 (11)	
H24A	0.1480	0.4510	0.5501	0.085*	
H24B	0.2838	0.4396	0.6396	0.085*	
N1	0.2484 (3)	0.5572 (2)	0.5960 (2)	0.0669 (8)	
H1C	0.1994	0.5750	0.6287	0.100*	
N2	0.2616 (3)	0.49323 (18)	0.41684 (19)	0.0567 (7)	
H2A	0.3047	0.4879	0.3813	0.085*	
Br2	0.41434 (4)	0.63994 (3)	1.24310 (3)	0.07881 (17)	
O2	−0.0266 (2)	0.59459 (14)	0.65772 (15)	0.0587 (6)	
H2B	−0.0912	0.5604	0.6430	0.088*	
C11	0.1170 (3)	0.65519 (19)	0.8032 (2)	0.0436 (7)	
H11A	0.1287	0.7005	0.7670	0.052*	
C12	0.0343 (3)	0.59134 (18)	0.7567 (2)	0.0429 (7)	
C13	0.0169 (3)	0.52264 (18)	0.8108 (2)	0.0470 (8)	
H13A	−0.0407	0.4797	0.7795	0.056*	
C14	0.0843 (3)	0.51849 (18)	0.9094 (2)	0.0450 (7)	
H14A	0.0734	0.4720	0.9441	0.054*	
C15	0.1700 (3)	0.58307 (17)	0.9598 (2)	0.0388 (7)	
C16	0.2394 (3)	0.58006 (19)	1.0613 (2)	0.0458 (8)	
H16A	0.2318	0.5338	1.0973	0.055*	
C17	0.3176 (3)	0.6450 (2)	1.1062 (2)	0.0489 (8)	
C18	0.3303 (3)	0.7159 (2)	1.0545 (2)	0.0533 (8)	
H18A	0.3826	0.7603	1.0873	0.064*	
C19	0.2665 (3)	0.71977 (19)	0.9567 (2)	0.0499 (8)	
H19A	0.2765	0.7668	0.9225	0.060*	
C20	0.1845 (3)	0.65347 (18)	0.9052 (2)	0.0395 (7)	

### Atomic displacement parameters (Å^2^)

**Table:**

	*U*^11^	*U*^22^	*U*^33^	*U*^12^	*U*^13^	*U*^23^
Br1	0.0786 (3)	0.0808 (3)	0.0568 (3)	−0.0023 (2)	0.0141 (2)	−0.0183 (2)
O1	0.0537 (13)	0.0915 (19)	0.0459 (14)	0.0005 (13)	0.0124 (11)	−0.0070 (13)
C1	0.049 (2)	0.055 (2)	0.054 (2)	0.0111 (16)	0.0221 (17)	0.0048 (16)
C2	0.0456 (19)	0.054 (2)	0.049 (2)	0.0120 (16)	0.0124 (16)	0.0006 (16)
C3	0.0442 (18)	0.060 (2)	0.053 (2)	0.0006 (16)	0.0131 (16)	−0.0032 (17)
C4	0.0430 (18)	0.058 (2)	0.060 (2)	0.0009 (16)	0.0223 (16)	0.0040 (17)
C5	0.0406 (17)	0.0422 (18)	0.055 (2)	0.0082 (15)	0.0182 (15)	0.0014 (15)
C6	0.051 (2)	0.053 (2)	0.055 (2)	0.0066 (17)	0.0208 (17)	−0.0032 (17)
C7	0.052 (2)	0.047 (2)	0.053 (2)	0.0073 (16)	0.0131 (17)	−0.0075 (16)
C8	0.051 (2)	0.048 (2)	0.069 (2)	0.0036 (16)	0.0162 (18)	−0.0050 (17)
C9	0.050 (2)	0.054 (2)	0.069 (2)	0.0040 (17)	0.0269 (18)	0.0024 (18)
C10	0.0386 (17)	0.0399 (18)	0.057 (2)	0.0070 (14)	0.0179 (15)	0.0027 (15)
C21	0.0451 (19)	0.072 (3)	0.073 (3)	0.0027 (18)	0.0160 (18)	−0.006 (2)
C22	0.0477 (19)	0.067 (2)	0.051 (2)	−0.0040 (18)	0.0116 (16)	0.0067 (17)
C23	0.0415 (18)	0.061 (2)	0.072 (2)	−0.0067 (17)	0.0090 (17)	−0.0002 (19)
C24	0.045 (2)	0.107 (3)	0.056 (2)	−0.016 (2)	0.0079 (17)	0.021 (2)
N1	0.0517 (17)	0.103 (3)	0.0512 (18)	−0.0028 (17)	0.0240 (14)	−0.0125 (18)
N2	0.0468 (15)	0.073 (2)	0.0480 (16)	−0.0084 (15)	0.0109 (13)	−0.0097 (15)
Br2	0.0957 (3)	0.0887 (3)	0.0446 (2)	−0.0178 (2)	0.0114 (2)	−0.00418 (19)
O2	0.0544 (13)	0.0687 (16)	0.0464 (14)	−0.0138 (12)	0.0064 (11)	0.0010 (11)
C11	0.0386 (17)	0.0419 (18)	0.0513 (19)	0.0011 (14)	0.0153 (15)	0.0094 (15)
C12	0.0347 (16)	0.047 (2)	0.0464 (19)	0.0035 (15)	0.0110 (14)	0.0000 (15)
C13	0.0427 (17)	0.0382 (18)	0.056 (2)	−0.0048 (14)	0.0095 (15)	−0.0056 (15)
C14	0.0477 (17)	0.0337 (17)	0.054 (2)	0.0003 (15)	0.0163 (15)	0.0046 (15)
C15	0.0359 (16)	0.0357 (17)	0.0458 (18)	0.0038 (13)	0.0141 (14)	0.0001 (14)
C16	0.0447 (18)	0.0460 (19)	0.049 (2)	0.0014 (15)	0.0182 (15)	0.0042 (15)
C17	0.0495 (18)	0.056 (2)	0.0416 (18)	0.0002 (16)	0.0151 (15)	−0.0034 (16)
C18	0.057 (2)	0.049 (2)	0.055 (2)	−0.0079 (16)	0.0181 (17)	−0.0094 (16)
C19	0.057 (2)	0.0390 (19)	0.054 (2)	−0.0082 (15)	0.0184 (17)	−0.0020 (15)
C20	0.0337 (15)	0.0402 (18)	0.0458 (18)	0.0044 (13)	0.0144 (14)	0.0004 (14)

### Geometric parameters (Å, °)

**Table:**

Br1—C7	1.896 (3)	C23—C24	1.490 (5)
O1—C2	1.371 (4)	C23—H23A	0.9700
O1—H1B	0.8168	C23—H23B	0.9700
C1—C2	1.353 (4)	C24—N1	1.460 (5)
C1—C10	1.427 (4)	C24—H24A	0.9700
C1—H1A	0.9300	C24—H24B	0.9700
C2—C3	1.405 (4)	N1—H1C	0.8336
C3—C4	1.361 (4)	N2—H2A	0.7730
C3—H3A	0.9300	Br2—C17	1.903 (3)
C4—C5	1.392 (4)	O2—C12	1.360 (3)
C4—H4A	0.9300	O2—H2B	0.8329
C5—C10	1.423 (4)	C11—C12	1.369 (4)
C5—C6	1.431 (4)	C11—C20	1.408 (4)
C6—C7	1.338 (4)	C11—H11A	0.9300
C6—H6A	0.9300	C12—C13	1.403 (4)
C7—C8	1.414 (5)	C13—C14	1.367 (4)
C8—C9	1.355 (4)	C13—H13A	0.9300
C8—H8A	0.9300	C14—C15	1.409 (4)
C9—C10	1.406 (4)	C14—H14A	0.9300
C9—H9A	0.9300	C15—C16	1.406 (4)
C21—N1	1.470 (4)	C15—C20	1.420 (4)
C21—C22	1.495 (4)	C16—C17	1.356 (4)
C21—H21A	0.9700	C16—H16A	0.9300
C21—H21B	0.9700	C17—C18	1.398 (4)
C22—N2	1.468 (4)	C18—C19	1.350 (4)
C22—H22A	0.9700	C18—H18A	0.9300
C22—H22B	0.9700	C19—C20	1.419 (4)
C23—N2	1.464 (4)	C19—H19A	0.9300
			
C2—O1—H1B	106.3	N2—C23—H23B	109.8
C2—C1—C10	120.2 (3)	C24—C23—H23B	109.8
C2—C1—H1A	119.9	H23A—C23—H23B	108.2
C10—C1—H1A	119.9	N1—C24—C23	109.2 (3)
C1—C2—O1	118.7 (3)	N1—C24—H24A	109.8
C1—C2—C3	120.3 (3)	C23—C24—H24A	109.8
O1—C2—C3	121.0 (3)	N1—C24—H24B	109.8
C4—C3—C2	120.8 (3)	C23—C24—H24B	109.8
C4—C3—H3A	119.6	H24A—C24—H24B	108.3
C2—C3—H3A	119.6	C24—N1—C21	110.0 (3)
C3—C4—C5	120.7 (3)	C24—N1—H1C	116.5
C3—C4—H4A	119.6	C21—N1—H1C	99.4
C5—C4—H4A	119.6	C23—N2—C22	110.9 (3)
C4—C5—C10	119.1 (3)	C23—N2—H2A	112.0
C4—C5—C6	122.4 (3)	C22—N2—H2A	104.1
C10—C5—C6	118.4 (3)	C12—O2—H2B	107.6
C7—C6—C5	120.3 (3)	C12—C11—C20	121.1 (3)
C7—C6—H6A	119.9	C12—C11—H11A	119.5
C5—C6—H6A	119.9	C20—C11—H11A	119.5
C6—C7—C8	121.4 (3)	O2—C12—C11	119.3 (3)
C6—C7—Br1	120.6 (3)	O2—C12—C13	121.0 (3)
C8—C7—Br1	118.0 (3)	C11—C12—C13	119.8 (3)
C9—C8—C7	119.7 (3)	C14—C13—C12	120.3 (3)
C9—C8—H8A	120.1	C14—C13—H13A	119.9
C7—C8—H8A	120.1	C12—C13—H13A	119.9
C8—C9—C10	121.2 (3)	C13—C14—C15	121.5 (3)
C8—C9—H9A	119.4	C13—C14—H14A	119.3
C10—C9—H9A	119.4	C15—C14—H14A	119.3
C9—C10—C5	118.9 (3)	C16—C15—C14	122.3 (3)
C9—C10—C1	122.3 (3)	C16—C15—C20	119.7 (3)
C5—C10—C1	118.8 (3)	C14—C15—C20	118.1 (3)
N1—C21—C22	109.2 (3)	C17—C16—C15	119.6 (3)
N1—C21—H21A	109.9	C17—C16—H16A	120.2
C22—C21—H21A	109.9	C15—C16—H16A	120.2
N1—C21—H21B	109.9	C16—C17—C18	121.7 (3)
C22—C21—H21B	109.9	C16—C17—Br2	119.5 (2)
H21A—C21—H21B	108.3	C18—C17—Br2	118.7 (2)
N2—C22—C21	109.8 (3)	C19—C18—C17	119.7 (3)
N2—C22—H22A	109.7	C19—C18—H18A	120.1
C21—C22—H22A	109.7	C17—C18—H18A	120.1
N2—C22—H22B	109.7	C18—C19—C20	121.2 (3)
C21—C22—H22B	109.7	C18—C19—H19A	119.4
H22A—C22—H22B	108.2	C20—C19—H19A	119.4
N2—C23—C24	109.4 (3)	C11—C20—C19	122.8 (3)
N2—C23—H23A	109.8	C11—C20—C15	119.3 (3)
C24—C23—H23A	109.8	C19—C20—C15	117.9 (3)
			
C10—C1—C2—O1	178.7 (3)	C22—C21—N1—C24	−60.3 (4)
C10—C1—C2—C3	−1.9 (5)	C24—C23—N2—C22	58.6 (3)
C1—C2—C3—C4	1.6 (5)	C21—C22—N2—C23	−58.0 (3)
O1—C2—C3—C4	−179.0 (3)	C20—C11—C12—O2	−178.6 (2)
C2—C3—C4—C5	−0.3 (5)	C20—C11—C12—C13	−0.4 (4)
C3—C4—C5—C10	−0.7 (5)	O2—C12—C13—C14	176.8 (3)
C3—C4—C5—C6	−178.7 (3)	C11—C12—C13—C14	−1.3 (4)
C4—C5—C6—C7	178.1 (3)	C12—C13—C14—C15	1.4 (4)
C10—C5—C6—C7	0.1 (4)	C13—C14—C15—C16	179.6 (3)
C5—C6—C7—C8	1.0 (5)	C13—C14—C15—C20	0.2 (4)
C5—C6—C7—Br1	−177.5 (2)	C14—C15—C16—C17	−178.3 (3)
C6—C7—C8—C9	−1.5 (5)	C20—C15—C16—C17	1.2 (4)
Br1—C7—C8—C9	177.0 (2)	C15—C16—C17—C18	0.6 (5)
C7—C8—C9—C10	0.9 (5)	C15—C16—C17—Br2	−178.8 (2)
C8—C9—C10—C5	0.2 (5)	C16—C17—C18—C19	−1.7 (5)
C8—C9—C10—C1	−178.9 (3)	Br2—C17—C18—C19	177.7 (2)
C4—C5—C10—C9	−178.8 (3)	C17—C18—C19—C20	1.0 (5)
C6—C5—C10—C9	−0.7 (4)	C12—C11—C20—C19	−177.4 (3)
C4—C5—C10—C1	0.3 (4)	C12—C11—C20—C15	2.0 (4)
C6—C5—C10—C1	178.4 (3)	C18—C19—C20—C11	−179.7 (3)
C2—C1—C10—C9	−179.9 (3)	C18—C19—C20—C15	0.8 (4)
C2—C1—C10—C5	1.0 (4)	C16—C15—C20—C11	178.7 (3)
N1—C21—C22—N2	58.1 (4)	C14—C15—C20—C11	−1.9 (4)
N2—C23—C24—N1	−59.7 (4)	C16—C15—C20—C19	−1.8 (4)
C23—C24—N1—C21	61.2 (3)	C14—C15—C20—C19	177.6 (3)

### Hydrogen-bond geometry (Å, °)

**Table:**

*D*—H···*A*	*D*—H	H···*A*	*D*···*A*	*D*—H···*A*
O1—H1B···N1	0.82	1.94	2.743 (4)	168
O2—H2B···N2^i^	0.83	1.88	2.694 (4)	163
N1—H1C···O2	0.83	2.47	3.235 (4)	152
N2—H2A···O1^ii^	0.77	2.50	3.184 (4)	149
C4—H4A···Cg5	0.93	2.77	3.471 (3)	133
C14—H14A···Cg2^iii^	0.93	2.68	3.371 (3)	132
C16—H16A···Cg1^iii^	0.93	2.90	3.570 (3)	130
C21—H21A···Cg2^iv^	0.97	2.93	3.831 (3)	156

Symmetry codes: (i) −*x*, −*y*+1, −*z*+1; (ii) −*x*+1, −*y*+1, −*z*+1;  (iii) −*x*+1, −*y*+1, −*z*+2; (iv) *x*−3/2, −*y*+1/2, *z*−3/2.
